# Sustainable Bamboo-Based Magnetic Activated Carbon for Adsorption of Cationic and Anionic Dyes from Wastewater: Kinetics, Isotherms, and Thermodynamics

**DOI:** 10.3390/ma19102110

**Published:** 2026-05-17

**Authors:** Asif Ali, Michiaki Matsumoto, Yoshiro Tahara, Shahzad Khan, Abbas Ali, Atta Ur Rahman

**Affiliations:** 1 Department of Applied Chemistry, Graduate School of Science and Engineering, Doshisha University, Kyotanabe 610-0321, Japan; asifaliawkum2@gmail.com (A.A.); ytahara@mail.doshisha.ac.jp (Y.T.); 2 School of Chemistry and Chemical Engineering, Beijing Institute of Technology, Beijing 100081, China; shahzad191213@gmail.com; 3 Department of Physics and Chemistry, Daegu Gyeongbuk Institute of Science and Technology, Daegu 42988, Republic of Korea; abbasali@dgist.ac.kr; 4 Asian Institute of Medical and Management Science, Khyber Medical University, Peshawar 25100, Pakistan; attaurrahmangeo@gmail.com

**Keywords:** magnetic activated carbon, bamboo biomass, dyes adsorption, wastewater treatment, adsorption isotherms

## Abstract

This study presents the synthesis and use of a novel bamboo-derived magnetic activated carbon (BMAC) for the effective removal of cationic and anionic dyes, specifically methylene blue (MB), methyl orange (MO), and sunset yellow (SY), from aqueous solutions. The adsorbent was synthesized using thermal carbonization and subsequent inclusion of magnetic oxide, yielding a porous structure with improved adsorption and magnetic separation properties. Thorough characterization utilizing SEM, EDX, BET, FTIR, XRD, and TGA/DTA validated the creation of a highly porous material including uniformly dispersed magnetic particles and several surface functional groups. Batch adsorption tests were performed to examine the influences of contact time, adsorbent dosage, initial dye concentration, pH, and temperature. The findings indicated rapid adsorption kinetics, with equilibrium reached in around 60–70 min, and adsorption capacity ranked as MB > MO > SY. Augmenting adsorbent dosage enhanced removal efficiency but diminished adsorption capacity per unit mass due to site unsaturation. The maximum adsorption capacities (q_m_) of BMAC were 58.9, 56.3, and 32.7 mg/g for MB, MO, and SY, respectively, as determined from the Langmuir isotherm model, indicating superior performance compared with other reported magnetic activated carbon. The adsorption process was determined to be exothermic and spontaneous, as evidenced by thermodynamic characteristics. The equilibrium data were optimally characterized by the Langmuir isotherm model, indicating monolayer adsorption, whereas the kinetic studies conformed to the pseudo-second-order model, signifying that chemisorption is predominant. The adsorption mechanism encompasses electrostatic interactions, π–π stacking, hydrogen bonding, van der Waals forces, pore filling, and surface complexation with magnetic oxides. The findings indicate that BMAC is an efficient, sustainable, and magnetically recoverable adsorbent for the elimination of both cationic and anionic dyes from wastewater.

## 1. Introduction

The discharge of synthetic dyes from diverse industrial activities has emerged as a significant global environmental concern about water contamination. Synthetic dyes, widely utilized in industries like textiles, food, cosmetics, and pharmaceuticals, are detrimental to human health and pose significant risks to aquatic ecosystems. Numerous dyes exhibit toxicity, persistence, and non-biodegradability, making their removal from wastewater. The risk of bioaccumulation and detrimental impacts on aquatic creatures highlights the pressing necessity for effective removal solutions [1,2]. Moreover, these dyes are generally resistant to natural degradation, resulting in their accumulation in aquatic environments, which adversely impacts human health and environmental integrity [3,4]. Traditional removal techniques, including chemical coagulation, flocculation, membrane filtration, and oxidation processes, frequently entail elevated operational expenses, inefficiencies, and considerable environmental hazards [5,6]. These limitations highlight the need for cost-effective, sustainable and environmental friendly treatment technologies for dye-contaminated wastewater. Among the numerous technologies available, adsorption has surfaced as a viable choice owing to its simplicity, cost-effectiveness, and high efficacy [7,8]. Conventional adsorbents, including activated carbon, are extensively utilized for dye elimination; nonetheless, their elevated production expenses, dependence on ecologically detrimental activation techniques, and restricted regeneration capacity constrain their broader utilization [9,10].

There has been a growing interest in biomass-derived adsorbents, such as biochar, sourced from renewable organic materials, as a sustainable approach to wastewater treatment. Bamboo-derived AC has attracted interest owing to its elevated carbon content, fast growth, and plentiful availability. Bamboo-derived AC demonstrates superior adsorptive characteristics, encompassing a substantial surface area, considerable porosity, and an array of functional groups that enhance dye elimination [11,12]. Bamboo-derived AC can be readily transformed via pyrolysis and chemical treatment, which augment its adsorption capacity and reusability, rendering it a desirable and sustainable material for environmental rehabilitation [13,14].

Furthermore, the advancement of magnetic AC has garnered interest owing to its efficacy in eliminating harmful substances and its convenient retrieval via magnetic separation. In contrast to conventional recovery techniques like filtration or centrifugation, magnetic separation minimizes operational expenses and prevents the formation of secondary sludge [15,16]. Magnetic AC is produced by integrating iron oxide nanoparticles (such as Fe_3_O_4_ and Fe_2_O_3_), which enhance adsorption effectiveness and facilitate material regeneration, hence improving its economic viability for prolonged utilization [16,17,18]. Previous studies have demonstrated that magnetic AC composites can eliminate more than 99% of pollutants from actual wastewater samples, hence reinforcing its viability for extensive applications [18,19].

This study examines the production and utilization of a unique Bamboo Magnetic Activated Carbon (BMAC) specifically engineered for the elimination of synthetic colors from wastewater. The substance was synthesized using the thermal carbonization of bamboo biomass and the incorporation of magnetic oxides to augment its adsorptive capabilities. The adsorption capabilities of BMAC for the model dyes Methylene Blue (MB), Methyl Orange (MO), and Sunset Yellow (SY) were assessed under varying conditions, including solution pH, adsorbent dosage, initial dye concentration, and temperature. The kinetics and thermodynamics of the adsorption process were modeled to elucidate the adsorption mechanism, identify rate-limiting phases, and assess the energy profile of dye removal. This study illustrates the viability of utilizing agricultural waste as a premium adsorbent, fostering a more sustainable and economical method for industrial wastewater treatment.

## 2. Materials and Methods

### 2.1. Reagents

**Figure 1:**
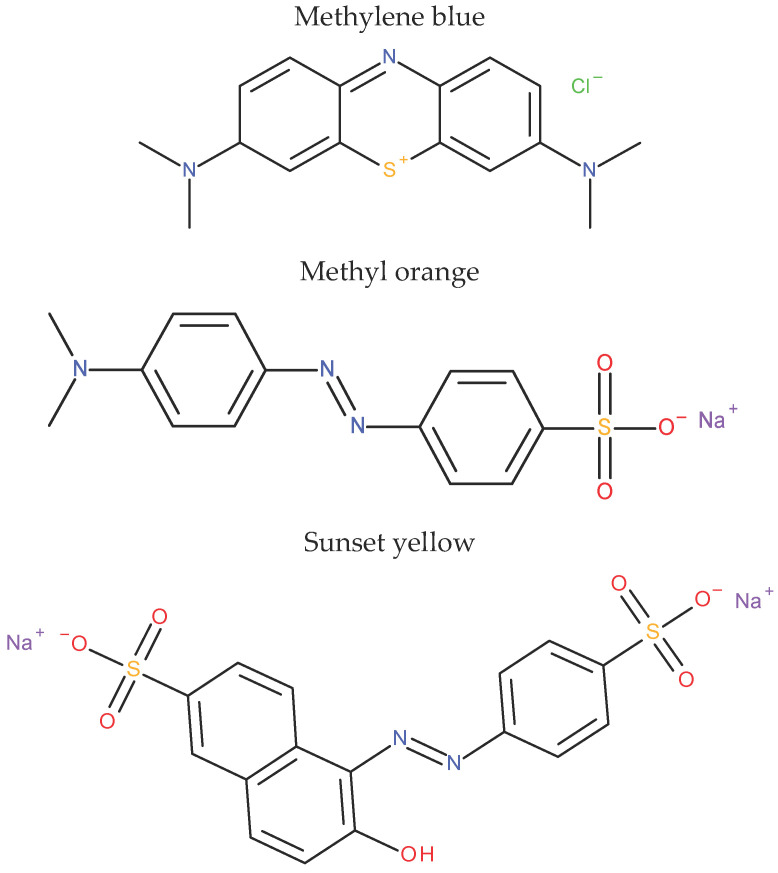
Chemical structures of the dyes used in this study.

All reagents used were of analytical grade and were as received without further purification. Stock solutions each with a concentration of 1000 mg/L were prepared by dissolving 1 g of MB, MO or SY in 1 L of distilled water in volumetric flasks. These solutions were subsequently diluted with distilled water to provide solutions having the desired concentrations of the dyes. The pH of each test solution was adjusted by adding 0.1 M NaOH or HCl solutions using a pH meter (F-7`, HORIBA, Kyoto, Japan). The concentrations of the dyes were measured at wavelength of 665, 464 and 482 nm for MB, MO and SY, respectively, using an UV-visible spectrophotometer (UV-2550, Shimadzu, Kyoto, Japan). The molecular structures of these dyes were shown in Figure 1.

### 2.2. Synthesis of Bamboo Magnetic Activated Carbon (BMAC)

Bamboo was collected from a local bamboo processing unit in Kyotanabe Kyoto, Japan and then washed with deionized water to remove dust and other impurities. The cleaned bamboo was dried in direct sunlight for 2 days, crushed into powder, and stored in a bottle. The BMAC was produced through chemical co-precipitation and thermal activation method. In a typical process, 12 g of ferric chloride (FeCl_3_) and 6 g of ferrous chloride hexahydrate (FeCl_2_·6H_2_O) was dissolved in 200 mL of distilled water with intensive stirring at 80 °C. Following this, 10 g of finely powdered bamboo was added to the solution and resulting dispersion was stirred for 1 h. A 20 mL of 25% NaOH solution was gradually added to this dispersion to form a black magnetic precipitate that was collected by filtration and then dried at 100 °C for 12 h. The dried product was heated in air at 650 °C for 3 h using a muffle furnace. The resulting activated carbon was cooled and then washed with deionized water until the resulting filtrate had a near-neutral pH (6–7). The final product was dried by heating at 110 °C in an oven for 6 h, after which it was stored for use in the adsorption experiments [20].

### 2.3. Adsorption Experiments

Batch adsorption experiments were performed to investigate the removal of MB, MO, and SY on BMAC. For isotherm studies, 100 mL of dye solution with concentrations ranging from 20 to 120 mg/L was placed in a glass vessel, and the pH was adjusted to the desired value using 0.1 M HCl or NaOH. A predetermined mass of BMAC (1–5 g/L) was added, and the mixture was stirred at room temperature (25 ± 1 °C) for 105 min. Equilibrium was confirmed when the dye concentration in solution showed no significant change over consecutive measurements. The residual dye concentration was determined using a UV-Vis spectrophotometer at 665 nm, 464 nm, and 482 nm for MB, MO, and SY, respectively. Adsorption isotherms were fitted using the Langmuir and Freundlich models in SigmaPlot 14.0.

Kinetic studies were conducted using an initial dye concentration of 100 mg/L, with 100 mL of solution and 1 g/L BMAC. The mixture was stirred at 25 °C, and samples were collected at 10, 20, 30, 40, 50, 60, and 70 min. After centrifugation, the supernatant was analyzed for residual dye concentration as above. The adsorption capacity at time t (q_t_, mg/g) was calculated using:

(1) $$q_{t}=\frac{\left(C_{0}-C_{t}\right)V}{m}$$

where *C*_0_ and *C*_t_ are the initial and time t concentrations (mg/L), *V* is the solution volume (L), and *m* is the mass of BMAC (g). Kinetic data were fitted to pseudo-first-order and pseudo-second-order models to determine the adsorption mechanism. All experiments were conducted in triplicate, and the average values are reported.

### 2.4. Instrumentation

The BMAC used in this work was characterized by several techniques. Fourier transform infrared (FT-IR) spectra (IRAffinity-1, MIRacle 10, Shimadzu Co., Kyoto, Japan) were acquired by the attenuated total reflectance (ATR) method. Brunauer–Emmett–Teller (BET) surface areas were determined using nitrogen adsorption (Belsorp mini, MicrotracBEL, Osaka, Japan). X-ray diffraction (XRD) analyses were performed on a RINT2000 instrument (Rigaku Corporation, Kyoto, Japan) using Cu Kα radiation. A JEOL JSM-7001FD microscope (JEOL Ltd., Akishima, Japan) was used to carry out detailed surface morphology examinations by scanning electron microscopy (SEM) with Energy-dispersive X-ray (EDX). Thermogravimetric analysis (TGA) and differential thermal analysis (DTA) were performed with a Shimadzu DTG 60 instrument.

## 3. Results

### 3.1. Characterization

**Figure 2:**
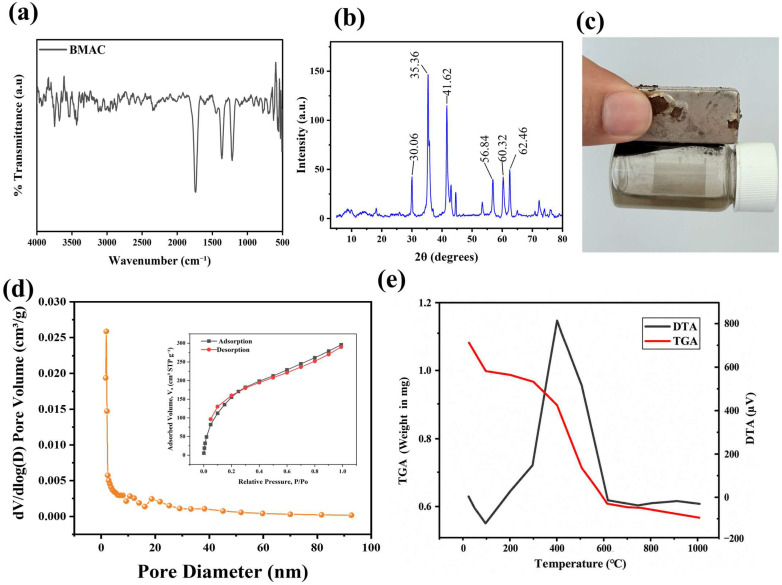
(**a**) Fourier transforms infrared spectroscopy, (**b**) X-ray diffraction, (**c**) Magnetic separation of BMAC in aqueous solution (**d**) Brunauer–Emmett–Teller analysis (**e**) thermogravimetric profile of BMAC.

The FTIR spectrum of BMAC (Figure 2a) displays a broad, low-intensity absorption band, indicating that the carbon matrix is largely amorphous. Although the characteristic peaks for hydroxyl (-OH), carbonyl (C=O), and carboxyl (-COOH) groups are not clearly visible, the surface is believed to contain a range of oxygen-containing functional groups. These groups, typically found in the wavenumber ranges of hydroxyl (-OH) at 3200–3550 cm^−1^, carbonyl (C=O) at 1650–1750 cm^−1^, and carboxyl (-COOH) around 2500–3300 cm^−1^, can promote hydrogen bonding and electrostatic interactions with dye molecules, thereby enhancing the material’s adsorption capabilities. However, the absence of distinct peaks for these functional groups in the FTIR spectrum can likely be attributed to the high-temperature pyrolysis and activation process, where many oxygen-containing groups such as -OH, C=O, and -COOH undergo thermal decomposition or elimination. At elevated temperatures (e.g., 500–700 °C), volatile components and unstable surface groups are released as gases like H_2_O, CO, and CO_2_, while the carbon matrix becomes more graphitized and amorphous, reducing the number of detectable oxygen functionalities by FTIR. This behavior is well-documented in the literature, where increasing carbonization/activation temperatures lead to a reduction in surface functional groups on biomass-derived carbons due to thermal degradation, despite their initial presence at lower temperatures. The broad absorption observed is typical of activated carbons derived from bamboo biomass, indicating a diverse set of surface functional groups that contribute to BMAC’s high adsorption efficiency [21,22,23,24].

The X-ray diffraction (XRD) pattern of Bamboo Magnetic Activated Carbon (BMAC) was obtained to examine the crystalline structure and phase composition of the material. The XRD spectrum, obtained from 5° to 80° 2θ, displays multiple significant peaks indicative of the material’s crystalline phases (Figure 2b). The pronounced peaks at around 30°, 35°, and 43° 2θ correspond to the diffraction planes of Fe_3_O_4_ (magnetite), a magnetic oxide frequently utilized in AC composites to improve adsorption characteristics [25]. Notably, there are no discernible peaks corresponding to graphitic or crystalline carbon (typically expected around 20–25°), indicating that the carbon in BMAC is largely amorphous or poorly ordered. The XRD pattern reveals a combination of crystalline and amorphous phases, demonstrating the effective integration of magnetic oxides into the bamboo-derived AC. The crystallinity level, along with the observed phases, substantiates the promise of BMAC for adsorption applications, particularly for cationic and anionic dye pollutants [26,27]. These findings align with prior research, indicating that activated carbon and magnetic AC composites demonstrate significant adsorption capacities attributable to their surface functional groups and magnetic characteristics [28].

The magnetic characteristics of BMAC were exhibited in an aqueous solution (Figure 2c). A vial with dispersed BMAC particles in water was positioned adjacent to a powerful neodymium magnet, causing the particles to swiftly migrate towards the magnet, resulting in a clear supernatant. This verifies that BMAC can be effectively isolated from the solution following dye adsorption, facilitating easy recovery and reutilization. The observed magnetic properties are ascribed to the integrated Fe_3_O_4_ nanoparticles.

The nitrogen (N_2_) adsorption–desorption isotherm at 77 K of BMAC is shown in Figure 2d. The adsorption curve exhibits a steep increase at low relative pressures (P/P_0_ < 0.1), indicating the presence of narrow micropores [29]. The desorption curve shows minimal hysteresis, consistent with the limited presence of larger pores. The small negative hysteresis observed at higher P/P_0_ may result from rapid desorption of nitrogen from narrow micropores. According to the IUPAC classification, the isotherm corresponds to Type I(b), confirming the narrow microporous nature of BMAC [30]. The BET surface area, calculated from the adsorption data, was 248.99 m^2^/g, with a total pore volume of 0.2083 cm^3^/g and an average pore diameter of 1.35 nm [31]. These results highlight the material’s potential for adsorption of small organic molecules, including dyes [27].

Thermogravimetric analysis (TGA) and differential thermal analysis (DTA) of BMAC were performed to examine its thermal stability, breakdown properties, and heat absorption characteristics (Figure 2e). The TGA curve indicates a two-step weight loss process: an initial weight reduction below 200 °C due to the elimination of moisture and light volatile organic compounds, followed by a substantial mass loss between 300 °C and 600 °C, signifying the decomposition of labile organic compounds and the development of a more stable carbonaceous structure. At temperatures beyond 600 °C, the weight loss stabilizes, signifying the existence of thermally stable carbon and magnetic oxides. The DTA curve enhances the TGA findings, revealing endothermic peaks between 100 °C and 300 °C, which align with moisture evaporation and heat absorption during the decompositionof volatile constituents. A subsequent endothermic peak between 400 °C and 600 °C indicates the decomposition of carbonaceous organic matter. The comprehensive profile demonstrates that BMAC maintains its structural integrity at high temperatures, affirming its suitability as a stable material for elevated temperature applications, including adsorption operations. The integration of TGA and DTA indicates that the inclusion of magnetic oxides improves the material’s thermal stability, rendering it appropriate for adsorption and remediation tasks in high-temperature environments. These findings align with research on magnetic AC materials, which exhibit enhanced thermal stability and adsorption effectiveness owing to the synergistic interaction between AC and magnetic modifications [30,32,33,34].

**Figure 3:**
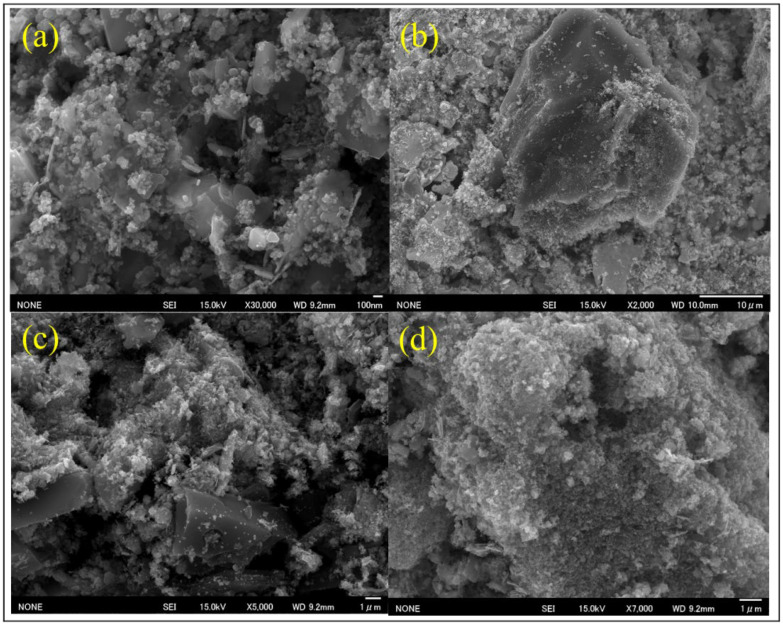
SEM micrographs of BMAC: (**a**) ×30,000 showing nanoscale roughness and irregular aggregates; (**b**) ×2000 showing larger macrostructures and smaller aggregates forming a hierarchical pore network; (**c**) ×5000 and (**d**) ×7000 showing flake-like clusters and interconnected pores.

**Figure 4:**
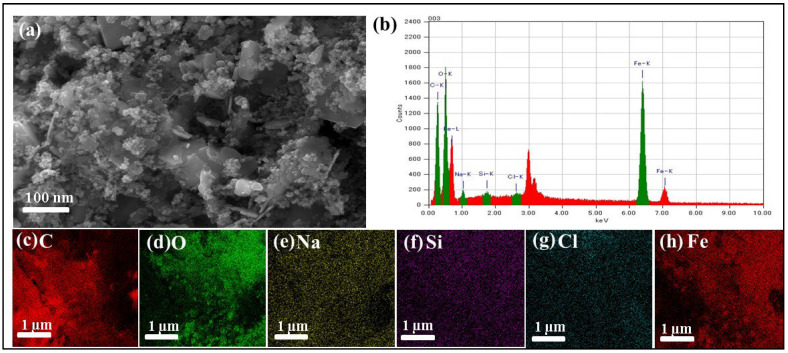
SEM and EDX analysis of BMAC: (**a**) SEM image of the area analyzed by EDX, (**b**) EDX spectrum showing major elements C, O, and Fe, with minor elements Na, Si, and Cl, and (**c**–**h**) Elemental mapping images showing the distribution of (**c**) C, (**d**) O, (**e**) Na, (**f**) Si, (**g**) Cl, and (**h**) Fe (the range of voltage described in (**c**–**h**) were highlighted as green in (**b**)), confirming the homogeneous dispersion of magnetic nanoparticle.

The surface morphology of BMAC was examined via scanning electron microscopy (SEM) at various magnifications (Figure 3 and Figure 4a). At high magnification (Figure 3a, ×30,000), the surface displays nanoscale roughness and uneven aggregates, suggesting a significant surface area and numerous active adsorption sites [31,35]. Images at lower magnification (Figure 2b, ×2000) display larger particle clusters interspersed with smaller aggregates, creating a hierarchical pore network [36,37]. Intermediate magnifications (Figure 3c, ×5000; Figure 3d, ×7000) reveal flake-like aggregates and interlinked pores, facilitating the movement of dye molecules and improving adsorption efficacy [38]. Figure 4a, the SEM picture associated with the EDX analysis region, exhibits analogous morphological attributes, hence affirming consistent surface properties throughout the sample [3,39].

Energy-dispersive X-ray (EDX) analysis of the same region (Figure 4b) confirms the elemental composition of BMAC. The spectrum indicates that the material is primarily composed of carbon (C) and oxygen (O), and the presence of iron (Fe) peaks validates the successful incorporation of magnetic nanoparticles. Quantitative surface percentages were not determined; however, the EDX results qualitatively confirm the expected elemental composition [36,40]. Trace elements including sodium (Na), silicon (Si), and chlorine (Cl) were found, presumably derived from the bamboo precursor or the synthesis process [41]. Elemental mapping (Figure 4c–h) illustrates a uniform distribution of these elements, especially Fe, which facilitates effective magnetic recovery and reliable adsorption performance [42,43,44].

The SEM and EDX investigations substantiate that BMAC exhibits a highly porous, rough, and compositionally homogeneous structure, integrating a high surface area with magnetic properties. These attributes render it a potential adsorbent for the effective elimination of cationic and anionic dyes from aqueous solutions.

### 3.2. Adsorption Study

The adsorption of MB, MO, and SY on BMAC was investigated under varying experimental conditions, including temperature; contact time, adsorbent dose, dye concentration, and solution pH. The interactions between BMAC and the dyes are primarily driven by surface adsorption, facilitated by hydroxyl (-OH), carbonyl (C=O), carboxyl (-COOH), and phenolic (-OH) functional groups present on the BMAC surface. These groups enable hydrogen bonding, electrostatic interactions, and π–π stacking, while the amine groups in MB and sulfonic acid groups in MO further enhance adsorption through electrostatic interactions with the adsorbent [45,46].

#### 3.2.1. Effect of Temperature on Adsorption

**Figure 5:**
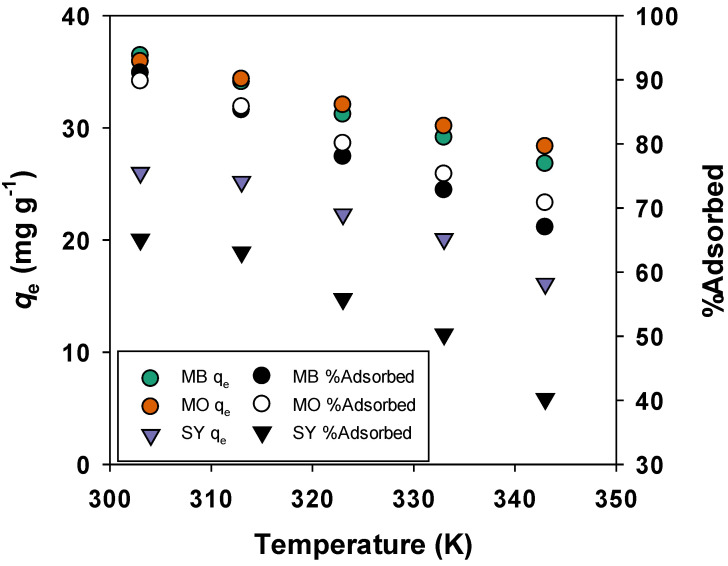
Effect of temperature on adsorption (Initial dye concentrations were 100 mg/L).

The effect of temperature on dye adsorption was examined within the temperature range of 303–343 K, as shown in Figure 5. The initial concentrations of the dyes used in the temperature-dependent experiments were 100 mg/L for each dye. This concentration was chosen to evaluate the adsorption capacity under consistent conditions across the specified temperature range.

It was observed that as temperature increased, the adsorption efficiency of the dyes decreased, indicating that the adsorption process is exothermic in nature. Specifically, the adsorption capacity for MB decreased from 36.45 mg/g (91.1% removal) at 303 K to 26.8 mg/g (67% removal) at 343 K. Similarly, the adsorption capacity for MO decreased from 35.93 mg/g (89.8% removal) to 28.35 mg/g (70.8% removal), and for SY, the adsorption capacity decreased from 26 mg/g (65.1% removal) to 16.1 mg/g (40.2% removal). These trends clearly suggest that lower temperatures favor dye adsorption, with MB exhibiting the greatest sensitivity to temperature variations.

By maintaining a consistent initial dye concentration of 100 mg/L, we were able to ensure that the results could be compared effectively across the temperature range, providing a reliable basis for assessing the influence of temperature on dye adsorption efficiency.

#### 3.2.2. Effect of Stirring Time on Adsorption

**Figure 6:**
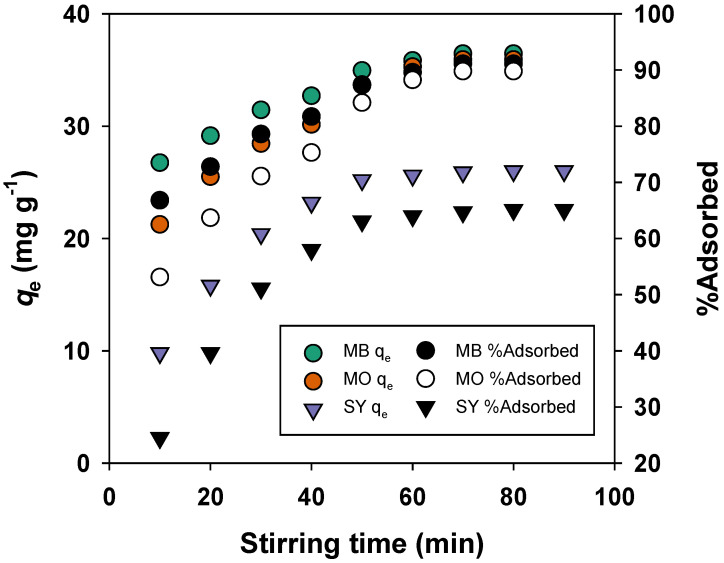
Effect of stirring time on adsorption efficiency.

The effect of stirring time on the adsorption of MB, MO, and SY onto BMAC is shown in Figure 6. As stirring time increased, the adsorption capacity of MB rose from 26.75 mg/g at 10 min to 36.45 mg/g at 70 min, while the removal efficiency increased from 66.8% to 91.1%, indicating that equilibrium was reached around 70 min. For MO, the adsorption capacity increased from 21.25 mg/g to 35.93 mg/g, and the removal efficiency rose from 53.1% to 89.8% by the 70 min mark. Similarly, SY adsorption increased from 9.8 mg/g to 26.0 mg/g, with the corresponding removal efficiency rising from 24.5% to 65.1% over the same period.

Overall, MB showed the highest adsorption capacity and removal efficiency, followed by MO and SY. The increase in adsorption with stirring time can be attributed to the progressive occupation of available active sites on the BMAC surface, with adsorption reaching equilibrium as the surface sites became saturated with dye molecules.

#### 3.2.3. Effect of Adsorbent Dose on Adsorption

**Figure 7:**
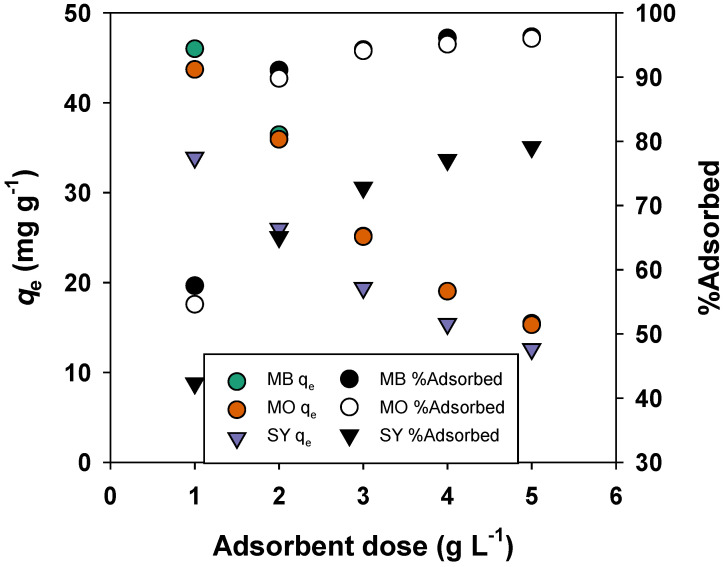
Effect of adsorbent dose on adsorption performance (70 min contact time, pH 7, initial dye concentration of 50 mg L^−1^, and temperature of 298 K.

The effect of adsorbent dosage on the adsorption performance of MB, MO, and SY onto BMAC is presented in Figure 7. As the adsorbent dosage increased from 1 to 5 g L^−1^, the removal efficiency of MB significantly increased from 57.5% to 96.3%, while the corresponding adsorption capacity decreased from 46.0 mg g^−1^ to 15.45 mg g^−1^. A similar trend was observed for MO, where the adsorption capacity decreased from 43.7 mg g^−1^ to 15.3 mg g^−1^, accompanied by an increase in removal efficiency from 54.6% to 96.0%. Likewise, SY showed a reduction in adsorption capacity from 33.9 mg g^−1^ to 12.6 mg g^−1^, whereas its removal efficiency increased from 42.3% to 79.1%.

The improvement in removal efficiency with increasing adsorbent dosage is attributed to the greater availability of active adsorption sites. In contrast, the decrease in adsorption capacity per unit mass at higher dosages may be due to incomplete utilization of active sites and possible aggregation of adsorbent particles, which reduces the effective surface area available for adsorption.

#### 3.2.4. Effect of Dye Concentration on Adsorption

**Figure 8:**
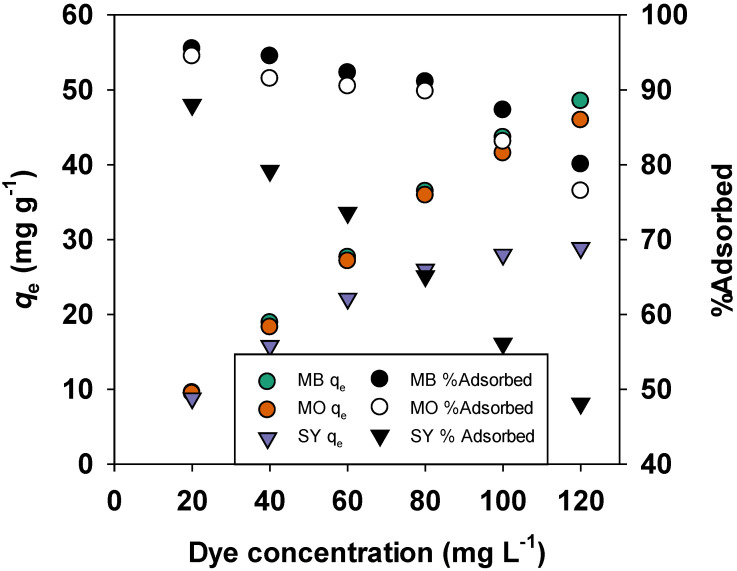
Effect of concentration of MB, MO, and SY dyes on adsorption.

The effect of increasing dye concentrations on the adsorption of MO, SY, and MB was assessed (Figure 8). As the dye concentration increased, the adsorption capacity of each dye also increased. Specifically, the adsorption capacity for MB rose from 9.55 mg/g at 20 mg/L to 48.5 mg/g at 120 mg/L. However, the proportion of MB adsorbed decreased from 95.5% to 80.08%, indicating that while more dye was adsorbed, the adsorption efficiency decreased as the active sites on BMAC became increasingly saturated. Similarly, MO’s adsorption capacity increased from 9.45 mg/g to 45.95 mg/g, but its proportion of adsorption decreased from 94.5% to 76.5%. SY adsorption showed an increase from 8.8 mg/g to 28.9 mg/g, with the proportion decreasing from 88% to 48.1%.

These results indicate that adsorption capacity improves at higher dye concentrations due to the increased availability of dye molecules. However, the adsorption efficiency decreases because the active sites on BMAC become saturated, limiting further increase in dye uptake despite the higher concentration.

#### 3.2.5. Effect of pH on Adsorption

**Figure 9:**
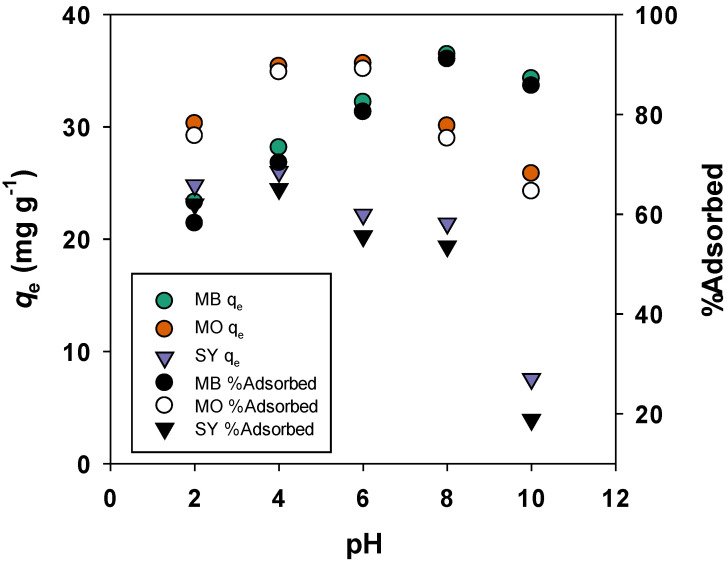
pH effect of adsorption.

The influence of solution pH on the adsorption of MO, SY, and MB onto BMAC is shown in Figure 9. The results indicate that the adsorption capacity of MB increases progressively with rising pH, reaching a peak near pH 8, before slightly decreasing at higher pH values. This trend suggests that the uptake of the cationic dye is most favorable under neutral to alkaline conditions, primarily due to stronger electrostatic interactions between the negatively charged BMAC surface and MB molecules.

In contrast, MO exhibits maximum adsorption at pH 4, with a noticeable decrease as pH increases. A similar trend is observed for SY, where adsorption is most significant under acidic conditions (pH 2–4), followed by a marked decline at higher pH levels.

The pHpzc of BMAC was determined to be 6.5, which is an important factor in understanding the surface charge behavior. At pH values above the pHpzc, the surface of BMAC becomes negatively charged, facilitating the adsorption of cationic dyes such as MB through electrostatic attraction. Below the pHpzc, the surface becomes positively charged, promoting the adsorption of anionic dyes (MO and SY) via electrostatic attraction. Thus, the adsorption process is strongly influenced by both the solution pH and the ionic characteristics of the dyes.

### 3.3. Theoretical Investigation

#### 3.3.1. Thermodynamics Analysis

**Figure 10:**
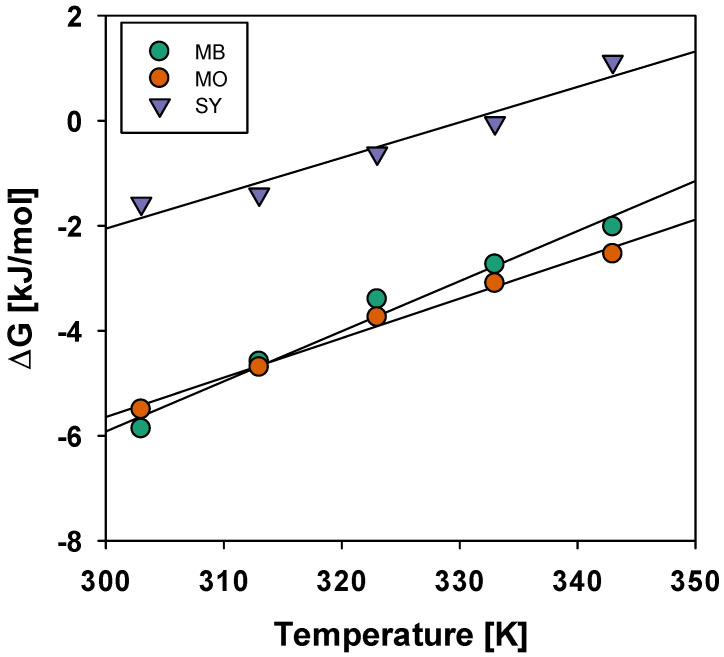
Van’t Hoff plots of ΔG° vs. temperature for MB, MO, and SY adsorption on BMAC. The plots were used to determine ΔH° and ΔS° for each dye.

In Figure 10 and Table 1 the thermodynamic parameters including Gibbs free energy (ΔG°), enthalpy (ΔH°) and entropy (ΔS°) were evaluated to investigate the adsorption behavior of MB, MO and SY dyes onto BMAC at different temperatures (303–343 K).

The equilibrium constant (*K*) was defines as

(2) $$K=\frac{q_{e}\cdot (adsorbent\;dose)}{C_{e}}\;$$

where *q*_e_ (mg g^−1^) is the adsorption capacity at equilibrium and *C*_e_ (mg L^−1^) is the equilibrium dye concentration remaining in solution.

The adsorption capacity *q*_e_ was determined using Equation (3):

(3) $$q_{e}=\frac{\left(C_{0}-C_{e}\right)V}{m}\;$$

where *C*_0_ and *C*_e_ are the initial and equilibrium dye concentrations (mg L^−1^), respectively, *V* is the volume of dye solution (L), and *m* is the mass of adsorbent (g).

For each initial dye concentration and temperature, the equilibrium dye concentration (*C*_e_) was experimentally determined after adsorption equilibrium was reached, and the corresponding equilibrium constant (*K*) was calculated individually.

The Gibbs free energy change was calculated using:

(4) ΔG°=−RTIn K

The relationship between thermodynamic parameters is expressed by the Van’t Hoff equation:

(5) ΔG°=ΔH°−TΔS°

#### 3.3.2. Isotherm Study

**Figure 11:**
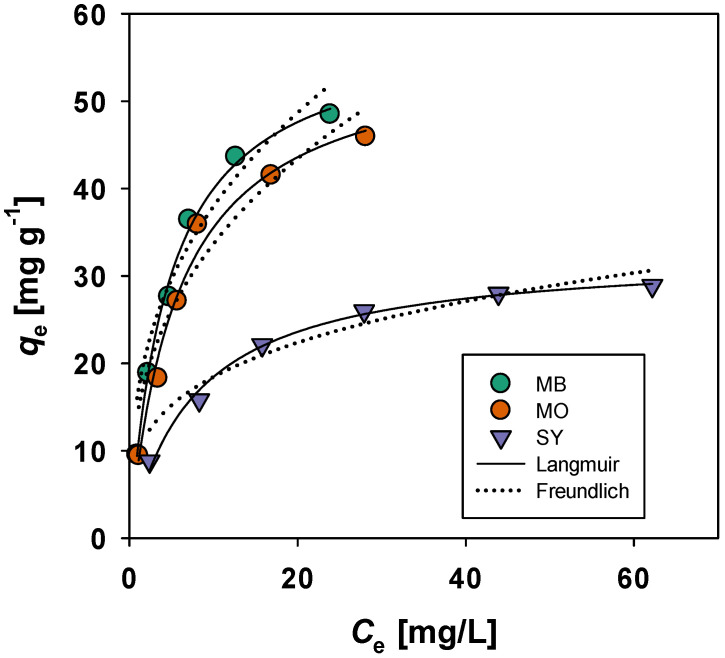
Langmuir and Freundlich adsorption isotherms for MB, MO, and SY adsorption on BMAC at different temperatures under identical experimental conditions (contact time = 70 min, initial dye concentration = 20–120 mg L^−1^, adsorbent dose = 5 g L^−1^, and optimized pH conditions for each dye).

To further elucidate the mechanism of dye adsorption, the Langmuir and Freundlich adsorption isotherms were used to analyze the experimental data for MB, MO, and SY adsorption on BMAC. The Langmuir model is typically used to describe monolayer adsorption, while the Freundlich model suggests multilayer adsorption on heterogeneous surfaces. The Langmuir model, which assumes uniform adsorption sites, is represented by the equation:

(6) $$q_{e}=\frac{q_{m}K_{L}C_{e}}{1+K_{L}C_{e}}$$

where $q_{m}$ is the maximum adsorption capacity (mg/g) and $K_{L}$ is the Langmuir adsorption constant (L/mg). This model assumes monolayer adsorption on a surface with no interaction between adsorbed molecules. The Freundlich model, which is suitable for multilayer adsorption and heterogeneous surfaces, is represented by the equation:

(7) $$q_{e}=K_{F}C_{e}^{\frac{1}{n}}$$

where $q_{e}$ is the adsorption capacity at equilibrium (mg/g), $K_{F}$ is the Freundlich constant, and n is the heterogeneity factor, reflecting the adsorption intensity. Both models were applied to the experimental data, and R^2^ values were used to assess the best-fitting model. The adsorption isotherm plots at different temperatures together with Langmuir and Freundlich model fitting curves are presented in Figure 11 and the corresponding parameters are listed in Table 2. The *R*^2^ values demonstrate that the Langmuir model provided a better description of the adsorption process.

#### 3.3.3. Kinetic Study

**Figure 12:**
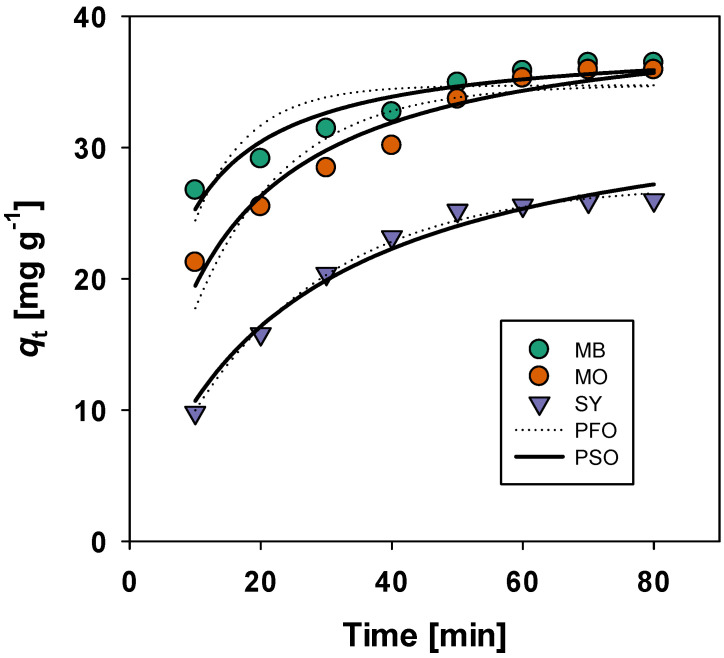
Change in the adsorption capacity (qt) with time. The dashed and solid lines were calculated using pseudo-first-order and pseudo-second-order kinetic models, respectively, under optimized experimental conditions (initial dye concentration = 100 mg/L, contact time = 10–80 min, adsorbent dose = X g/L, and optimized pH conditions for each dye).

The adsorption kinetics of MB, MO, and SY were analyzed using both the pseudo-first-order and pseudo-second-order models. The pseudo-first-order model assumes that the rate of adsorption is proportional to the difference between the equilibrium adsorption capacity and the amount adsorbed at time t. The equation for the pseudo-first-order (PFO) model is

(8) $$q_{t}=q_{e}\left(1-e^{-k_{1}t}\right)$$

where $k_{1}$ is the rate constant of the pseudo-first-order adsorption (min^−1^), $q_{t}$ is the amount adsorbed at time t (mg/g), and $q_{e}$ is the equilibrium adsorption capacity (mg/g). The pseudo-second-order (PSO) model assumes that the rate of adsorption is proportional to the square of the number of empty sites available for adsorption. The associated equation is

(9) $$q_{t}=\frac{k_{2}q_{e}^{2}t}{1+k_{2}q_{e}t}$$

where $k_{2}$ is the rate constant of the pseudo-second-order adsorption (g/(mg·min)). The kinetic parameters of each model calculated by the nonlinear least squares method included in Sigma Plot (version 14) are summarized in Table 3 while the resulting fits for each model are shown in Figure 12. This software was able to directly determine the parameters for Equations (3) and (4) without the need to generate linear plots. The PSO model provided a better fit for the kinetic data, as indicated by the higher *R*^2^ values (0.912–0.978), compared with those produced using the PFO model (0.698–0.995). The equilibrium adsorption capacities ($q_{e}$) obtained from the PSO model were closer to the experimental data, indicating that the adsorption process was likely governed by chemisorption, which is common in dye adsorption.

## 4. Mechanistic Insights

**Figure 13:**
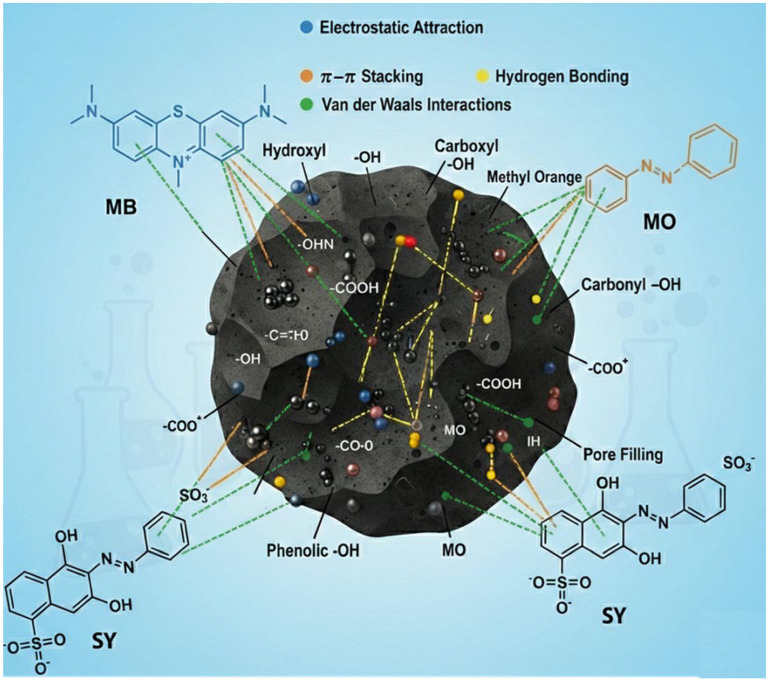
Schematic illustration of dyes adsorption with BMAC.

Figure 13 illustrates that the adsorption of dyes onto BMAC occurs via many synergistic mechanisms linked to its porous carbon structure, oxygen-containing surface functionalities, and magnetic oxide sites. The considerable surface area and advanced micro-/mesoporosity facilitate swift mass transfer and pore saturation, enabling MB, MO, and SY molecules to penetrate interior adsorption sites. Pore filling is widely acknowledged as a significant factor in dye adsorption by AC based adsorbents, in addition to surface interactions [16,47,48]. The aromatic configurations of these dyes can interact with the graphitized domains of the AC via π–π stacking, hence enhancing adsorption on the carbon surface [47,49]. The adsorption behavior is significantly influenced by the solution pH and the surface charge of BMAC. In negatively charged environments, deprotonated groups like –COO^−^ and –O^−^ promote the adsorption of the cationic dye MB via electrostatic attraction, while in acidic conditions, protonated groups such as –OH_2_^+^ and –COOH facilitate the uptake of anionic dyes like MO and SY [47,50]. Additionally, oxygen-containing functional groups on BMAC, such as hydroxyl, carboxyl, carbonyl, and phenolic groups, can engage in hydrogen bonding with dye molecules that possess –N, –N=N–, –SO_3_^−^, and –OH functionalities [49,51,52]. The Fe_3_O_4_ or other metal oxide sites on magnetic AC may offer supplementary adsorption centers via surface complexation and bridging interactions, so enhancing dye uptake and the recovery of the adsorbent through magnetic separation [53,54]. The synergistic effects of pore filling, π–π interactions, electrostatic attraction, hydrogen bonding, van der Waals forces, and surface complexation elucidate the quick kinetics, elevated adsorption capacities, and overall efficacy of BMAC for both cationic and anionic dyes [47,49,54].

## 5. Comparative Study

Magnetic Activated composites are agricultural wastes transformed into high dye removal efficiencies and adsorption capacities, which are generally between 80 and 350 mg/g. Magnetic separability is added by the incorporation of iron oxide nanoparticles (e.g., Fe_3_O_4_, -Fe_2_O_3_) which can also tend to boost adsorption and some materials have succeeded in removing more than 99% of impurities in real wastewater. Bamboo, rice husk, oil palm fronds and lignin find their way to be valorized into these adsorbents that prove to be quite versatile in the removal of cationic as well as anionic dyes [55,56,57]. For instance, Shao et al. have prepared magnetic biochar on solid waste through sewage sludge and iron tailings as dye (methylene blue) remover. The modified AC with iron exhibited improved adsorption capacity (82–91 mg g^−1^) which is mainly caused by the electrostatic attraction, pi-pi interactions, hydrogen bonding and surface complexation [58]. Gong et al. were able to develop a magnetic multifunction biochar based on cobs of corn and red mud to remove malachite green dye and Pb^2+^ in wastewater simultaneously. It was observed that the Activated Carbon possessed high adsorption capacities and it retained high removal efficiencies in the binary systems since site-specific adsorption processes were observed, with very little competition interference. In addition, the magnetic biochar demonstrated high regenerability, which is associated with the complex wastewater remediation [59]. In another study, Kapoor et al. made an iron and nitrogen co-doped AC of pineapple peel to remove Acid Yellow 23 dye, whereby maximum removal of up to 95% occurred with faster kinetics and less dosage than unfetched AC under acidic conditions [60]. Besides this, numerous magnetic bio-based adsorbents based on a variety of biomass sources have been reported in the literature and some of them are summarized in Table 4 with regard to their adsorption performance in wastewater treatment.

## 6. Conclusions

A unique bamboo-derived magnetic activated carbon (BMAC) was successfully synthesized and assessed as an effective adsorbent for the elimination of MB, MO, and SY from aqueous solutions. Characterization verified that BMAC features a highly porous architecture, numerous surface functional groups, and uniformly dispersed magnetic nanoparticles, all of which jointly improve adsorption efficacy and facilitate magnetic separation. Batch adsorption tests indicated that adsorption capacity augmented with contact time, achieving equilibrium within 60–70 min, whereas an increase in adsorbent dosage enhanced overall removal efficiency but diminished adsorption capacity per unit mass. The adsorption efficacy adhered to the sequence MB > MO > SY, indicating more robust interactions between the cationic dye and the negatively charged BMAC surface. BMAC exhibited high maximum adsorption capacities (58.9, 56.3, and 32.7 mg/g for MB, MO, and SY), demonstrating its effectiveness compared to previously reported magnetic AC sorbents for dye removal. Thermodynamic investigation indicated that the adsorption process is spontaneous and exothermic. The equilibrium data were optimally represented by the Langmuir isotherm model, signifying monolayer adsorption, but kinetic studies adhered to a pseudo-second-order model, implying the predominance of chemisorption. Mechanistic studies revealed that dye removal transpires via a confluence of electrostatic attraction, π–π stacking, hydrogen bonding, van der Waals forces, pore filling, and surface complexation with magnetic oxides, collectively elucidating the elevated adsorption efficiency and swift kinetics. BMAC exhibits significant potential as a cost-efficient, sustainable, and magnetically recoverable adsorbent for dye-contaminated wastewater, with bamboo biomass augmenting its environmental and economic viability, positioning it as a promising candidate for large-scale wastewater treatment applications.

## Figures and Tables

**Table 1:** Thermodynamic parameters for the adsorption of MB, MO, and SY dyes on BMAC at different temperatures.

	SY		MB		MO	
Temp. (K)	ΔG (kJ/mol)	Values of (K)	ΔG (kJ/mol)	Values of (K)	ΔG (kJ/mol)	Values of (K)
303	−1.572	1.867	−5.867	10.26	−5.5	8.876
313	−1.398	1.711	−4.591	5.837	−4.696	6.079
323	−0.618	1.259	−3.398	3.545	−3.743	4.031
333	−0.041	1.015	−2.736	2.686	−3.097	3.06
343	1.126	0.673	−2.019	2.03	−2.536	2.433
	**ΔH kJ/mol**	**ΔS k** **J** **/mol/k**	**ΔH kJ/mol**	**ΔS kJ/mol/k**	**ΔH kJ/mol**	**ΔS kJ/mol/k**
	−22.31	0.067	−34.57	−0.095	−30.21	−0.081

**Table 2:** Langmuir and Freundlich isotherm parameters for MB, MO, and SY adsorption on BMAC at different temperatures. The parameters were obtained from the model fitting curves shown in Figure 11 under identical experimental conditions (contact time = 70 min, initial dye concentration = 20–120 mg L^−1^, adsorbent dose = 5 g L^−1^, and optimized pH conditions for each dye).

	Langmuir			Freundlich		
	*K*_L_ [L/mg]	*q*_m_ [mg/g]	R^2^	1/*n*	*K*_F_	R^2^
MB	4.72 ± 0.41	58.9 ± 1.8	0.995	0.358 ± 0.01	16.7 ± 0.2	0.961
MO	5.84 ± 0.97	56.3 ± 3.3	0.983	0.367 ± 0.01	14.5 ± 0.2	0.963
SY	7.73 ± 0.83	32.7 ± 0.9	0.991	0.278 ± 0.01	9.72 ± 0.17	0.943

**Table 3:** Pseudo-first-order (PFO) and pseudo-second-order (PSO) kinetic parameters for MB, MO, and SY adsorption on BMAC. The parameters were obtained from the kinetic model fitting curves shown in Figure 12 under identical experimental conditions (initial dye concentration = 100 mg L^−1^, contact time = 10–80 min, adsorbent dose = 5 g L^−1^, and optimized pH conditions for each dye).

Dye	PFO			PSO		
	*k*_1_ (min^−1^)	*q*_e_ (mg/g)	R^2^	*k*_2_ (g mg^−1^ min^−1^)	*q*_e_ (mg/g)	R^2^
MB	(12.2 ± 2.0) × 10^−2^	34.7 ± 0.9	0.698	(5.14 ± 0.96) × 10^−3^	38.2 ± 0.9	0.912
MO	(7.1 ± 1.1) × 10^−2^	34.8 ± 1.2	0.857	(2.29 ± 0.37) × 10^−3^	40.5 ± 1.2	0.949
SY	(4.6 ± 0.2) × 10^−2^	27.2 ± 0.4	0.995	(1.27 ± 0.29) × 10^−3^	34.9 ± 2.0	0.978

**Table 4:** Various magnetic adsorbents and their dye removal efficiencies.

Feedstock	Magnetic Phase	Target Dye	Key Results (q_max_ or % Removal)	Ref.
Baobab Seeds	Fe_3_O_4_	Congo red	94.2%	[54]
Algae	Fe_3_O_4_	Azocarmine G2	71.3 mg/g	[31]
Rosa roxburghii	Fe_3_O_4_	Congo red	172.88 mg/g	[61]
Turmeric leaves	Fe_3_O_4_	MB, Congo red	323.625 mg g^−1^, 256.41 mg g^−1^	[62]
Furfural residue	Fe_3_O_4_	Congo red, Tetracycline, Bisphenol A	110.89 mg g^−1^, 602.81 mg g^−1^, 157.76 mg g^−1^	[63]
Lignin	Fe_3_O_4_	Congo red	94.3%	[64]
Coconut shell	Fe_3_O_4_	Bisphenol S	43.5 mg g^−1^	[65]
Rice husk	Copper-doped carbon dots loaded on magnetic biochar	Congo red	90.1%	[66]
Oil palm frond	Fe_3_O_4_	Crystal Violet, SY	149.03 mg g^−1^, 342.47 mg g^−1^	[67]
Bamboo	Fe_3_O_4_	MB, MO, SY	58.9 mg g^−1^, 56.3 mg g^−1^, 32.7 mg g^−1^	This study

## Data Availability

The original contributions presented in this study are included in the article. Further inquiries can be directed to the corresponding author.
